# Can the onset of heart failure be delayed by treating diabetic cardiomyopathy?

**DOI:** 10.1186/s13098-017-0219-z

**Published:** 2017-04-04

**Authors:** Anna Marcinkiewicz, Stanisław Ostrowski, Józef Drzewoski

**Affiliations:** 1 Department of Cardiac Surgery, Medical University of Lodz, Pomorska 251, 92-213 Lodz, Poland; 2 Department of Internal Diseases, Diabetology and Clinical Pharmacology, Medical University of Lodz, Lodz, Poland

**Keywords:** Diabetes mellitus, Cardiomyopathy, Coronary artery disease, Myocardial revascularization, Percutaneous intervention

## Abstract

The pathophysiology of diabetic cardiomyopathy (DC) is not fully understood. This frequently undiagnosed complication of chronic hyperglycemia leads to heart failure (HF). However, it is suggested that an appropriate metabolic control of diabetes at an early stage of this deleterious disease, is able to inhibit the development and progression of DC to HF. Recently, it has been postulated that myocardial ischaemia plays an important role in the development of this pathology. Results of the antianginal pharmacological treatment and revascularization are unsatisfactory and reveal a gap in our knowledge and current approaches to treating DC. Most recent studies emphasize the ischaemic component of DC as a key target for therapeutic strategies, which could change its unfavorable history. More stress is put on an early diagnosis of coronary artery disease (CAD), promoting prompt revascularization. Choosing the accurate time of surgical revascularization, with the inclusion of the metabolic background, can ensure complete revascularization with better prognosis. This review will focus on the complexity of DC and summarize contemporary knowledge of treatment strategies for patients with diabetes and CAD.

## Background

It is predicted that the number of Europeans with diabetes will increase from 52 million in 2011 to 64 million by 2030. According to the World Health Organization (WHO), the number of deaths due to diabetes will double between 2005 and 2030. Worldwide, nearly 3.4 million people annually die of a high blood glucose level. These terrifying statistics reveal our gap in knowledge and diagnostics, resulting in unsatisfactory results of the treatment. To change the unfavorable natural history of diabetes, perhaps we should begin with changing our approach to this complex entity. Interestingly, type 2 of this chronic disease (type 2 diabetes mellitus, T2DM) is definitely dominant and strongly associated with obesity, mainly visceral. Both of these metabolic abnormalities (diabetes + obesity = diabesity) are well-established independent risk factors of atherosclerosis and cardiovascular diseases, including coronary artery disease (CAD) and heart failure (HF) [1]. There is a general agreement that CAD and HF are diagnosed in a high proportion of diabetics. These two complications of diabetes are responsible for significantly higher mortality among this group of patients than in non-diabetics [2]. In autopsy reports, coronary arteries atherosclerosis was found in approximately 50% of diabetics [3]. Considering the extent and severity of coronary arteries atherosclerosis in people with diabetes, percutaneous intervention (PCI) is usually difficult or even impossible to perform. As a consequence, diabetics are frequently referred to surgical myocardial revascularization, and comprise about 1/3 of patients undergoing coronary artery by-pass grafting (CABG) [4]. It was shown that the risk of HF is increased two- to threefold in people with diabetes, compared with non-diabetic subjects. Currently, we do not have enough evidence that diabetic cardiomyopathy (DC) does not precede all cases of HF. Ischemic and nonischemic mechanisms are postulated to cause HF in diabetics. Complex CAD results in myocardial ischemia (ischemic mechanism). Nonischemic mechanism relies on an elevated blood glucose concentration and its consequences.

Recently, the dysfunction of coronary circulation is emphasised to contribute significantly in DC. As a consequence, CAD poses many more questions. At least two of them seem to be especially intriguing:

- do we have enough evidence to exclude the presence of CAD in patients diagnosed with DC?
- and when CAD actually sets in?

In this article, we discuss briefly the pathogenesis of diabetic cardiomyopathy, with a special focus on the role of CAD in the development and progression of this life-threatening complication. A novel element of the following review, is our thesis, concerning the definition of DC. We strongly believe that changing the time of revascularization in diabetics can slow down the progressive alterations in a heart muscle. Moreover, we present a current concept of individualized approach to the management of CAD in people with diabetes.

## The pathogenesis and definition of diabetic cardiomyopathy

Diabetic cardiomyopathy (DC) is defined as functional and structural abnormalities of myocardium in diabetics, without concomitant CAD and HA [5]. According to such definition, we assume that the cardiomyopathy is caused only by diabetes. However, currently the presence of DC raises a lot of controversy, and has become a field of intensive investigations [5]. The diagnosis of DC is almost impossible, when taking into account the high prevalence of HA, obesity and CAD in diabetics. According to data from observational studies [6], 80% of patients with T2DM have hypertension. As noted in a recent report, CAD may be found during coronary angiography even in 93.8% of diabetic individuals [7].

It was established that chronic hyperglycemia impairs the anatomy and function of ventricles and atria. Diabetic arteriopathy manifests itself as an increase in left atrium volume index (LAVI) [8]. Consequently, atrial fibrillation (AF) occurs more often in diabetics than in people without diabetes [9]. It was shown in the VALIANT study that LAVI >32 mL/m^2^ in diabetic individuals, after myocardial infarction, is a negative prognostic factor of mortality and hospitalization due to cardiovascular events in a 20-month follow-up [10].

## The ischemic mechanism of diabetic cardiomyopathy

**Fig. 1:**
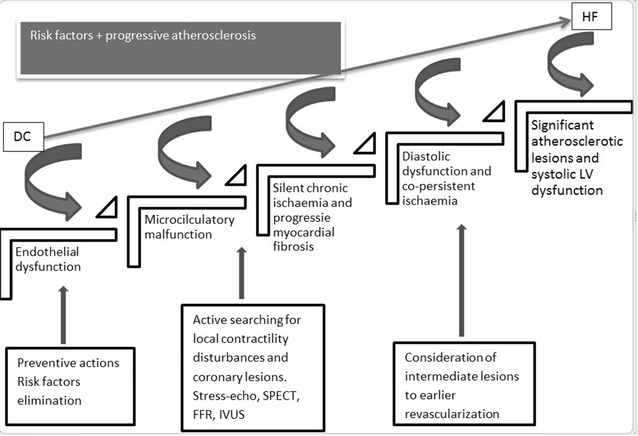
A scheme presenting continuity of ischaemic mechanism, leading from diabetic cardiomyopathy to heart failure. The upper part of the scheme includes additive factors, exacerbating the progress of diabetic cardiomyopathy. Therapeutic options are placed at the* bottom* of the scheme. *DC* diabetic cardiomyopathy, *HF* heart failure, *LV* left ventricle

The current insight into CAD puts emphasis on the microcirculatory dysfunction. An extensive body of literature exists on the impaired coronary microcirculation in diabetics, without epicardial arteries lesions, but depending on the endothelium impairment and its remodeling [11]. The prime consequence of these abnormalities is chronic myocardial ischemia, leading to fibrosis. Interstitial and perivascular fibrosis is the major histological finding in DC [5]. What’s more, fasting hyperglycaemia inhibits the formation of the coronary arteries collaterals [12]. Impaired arteriogenesis/angiogenesis predominantly depends on the loss of VEGF-stimulated monocytes migration and endothelial dysfunction. This phenomenon is an additive factor, promoting chronic ischemia (See Fig. 1). Figure 1 presents the above described, long-lasting process, beginning from endothelial dysfunction, being the cause of chronic myocardial ischaemia, followed by fibrosis and remodeling. This process directly results in diastolic and systolic cardiac dysfunction. Microvascular and macrovascular complications are often concomitantly present at the stage of diabetes diagnosis. Therefore, they considerably contribute to the development of clinical signs and symptoms of HF.

## The nonischemic mechanism of diabetic cardiomyopathy

Besides the ischaemic mechanism of diabetic cardiomyopathy, oxidative stress, increased susceptibility to ischemia/reperfusion injury, altered intracellular Ca^2+^ turnover, with reduced Ca^2+^ sensitivity of contractile proteins, the accumulation of advanced glycation end-products and cardiac autonomic neuropathy are among the postulated pathomechanisms of DC [5, 13]. Furthermore, atherogenic hyperlipidemia, FFA ectopic deposition, dysfunctional HDL without antioxidative, anti-inflammatory or vasodilative features, and diabetic thrombophilia make up the comprehensive presentation of a metabolic background, underlying full-symptomatic HF. Having in mind the multifactorial pathomechanism of DC, the following question arises: should the diabetic cardiomyopathy be redefined? Clinical practice indicates that the diagnosis of *pure* DC is almost impossible, because it was demonstrated that endothelial dysfunction develops even in prediabetes, which in turns leads to DC. Therefore, the evaluation of the coronary microcirculation could reveal patients with DC (if any) at a very early stage of this complication of chronic hyperglycaemia.

## Etiology of heart failure in diabetes

It is suggested that the etiology of HF is different in type 1 and 2 diabetes. Konduracka et al. [14] showed that myocardial dysfunction or HF was present only in patients with long-lasting type 1 diabetes, and only in the case of other co-existing cardiovascular risk factors (i.e. HA, CAD). Diabetes mellitus type 1 without co-existing cardiovascular risk factors did not cause myocardial dysfunction. So can we suspect that insulin deficiency and hyperglycaemia alone are not the cause of cardiomyopathy?

However, it is suggested that myocardial dysfunction, both in type 1 and 2 may develop, despite the lack of typical risk factors [5, 15]. Although in such situations, CAD is considered only as the presence of significant coronary stenosis/es, without taking into account the microcirculatory dysfunction.

## The influence of resistin on a heart muscle

Lebeche [16] presented an interesting conception about a high level of resistin, as a consequence of obesity, which can be a potential cause of insulin resistance and cardiac dysfunction. It was showed that resistin can impair heart contractility, increase oxidative stress, cause cardiomyocytes apoptosis, myocardial fibrosis and remodeling or cardiac hypertrophy [16–19]. It is also presumed that resistin can be involved in HA development, and impaired lipids metabolism in diabetic patients [16, 20, 21]. All of these facts would also explain the difference in etiology of HF, between diabetes type 1 and 2. What’s more, resistin is overexpressed locally, in an ischaemic myocardium [16, 22].

## New reports on pathogenesis of diabetic cardiomyopathy

Nutter et al. [23] recently suggested that high concentration of a protein RBFOX2 in cardiac cells can interfere with many important molecular mechanisms. In consequence, leading to inappropriate protein and calcium exchange in cardiomyocytes. On the other hand, Delbridge et al. [24] put emphasis on the elevated fructose concentration in diabetic patients, leading to increased fructose metabolism in cardiomyocytes, what results in unregulated glycolysis and oxidative stress. In turn, Li et al. [25] showed that impaired ubiquitin–proteasome system in mice with induced-diabetes caused proteotoxic stress and cardiomyopathy.

## A novel definition of diabetic cardiomyopathy

Given what is known about the relationship between CAD and DC, we propose a more accurate definition of DC. DC can be defined as a result of a long-lasting process, affecting the myocardium, that sets up, at a very early stage of metabolic changes (mainly associated with insulin resistance or resistin overexpression), even before diabetes is diagnosed, and soon after its beginning is accelerated by progressive myocardial ischemia (Fig. 1).

## Current standards of treatment in case of diabetes and coronary artery disease

Diabetic cardiomyopathy gradually progresses to HF, and despite pharmacological treatment, the prognosis remains poor. Therefore, appropriate multidirectional intervention at the stage of DC plays a key role in its progression to HF (See Fig. 1). It is suggested that revascularization in diabetics improves the prognosis of patients with diabetes, by inhibiting the HF onset [26]. Currently, the therapeutic strategy in diabetics with multivessel athelosclerotic lesions varies, depending on the CAD presentation. Stable CAD in diabetic individuals can be successfully managed with antianginal therapy [27]. Not only do angiotensin inhibitors (ACE-I, ARBs) ensure symptoms control, but also reduce myocardial fibrosis. Interestingly, candesartan was shown to limit collagen synthesis, and even promote its degradation, resulting in improved diastolic cardiac function [28].

## Timing of revascularization

Revascularization is frequently delayed in diabetic individuals. Usually patients with diabetes are referred to surgical revascularization at a stage, when coronary atherosclerosis is very severe, and many complications of diabetes are present. At such stage, HF is evident in a large portion of patients. What’s more, most of patients with diabetes aren’t referred directly to surgical revascularization after being diagnosed with CAD. Pandey et al. [29] draw attention to the fact that only one-third of diabetic patients admitted to hospital due to NSTEMI (non-STsegment elevation myocardial infarction) underwent surgical revascularization.

There are several clinical cases in which revascularization should be considered at an earlier stage of stable CAD: pharmacologically uncontrolled myocardial ischaemic symptoms, a large mass of myocardium threatened with ischaemia, left main stem (LMS) disease or proximal left anterior descending artery (LAD) stenosis, should accelerate revascularization [30]. What’s more, current cardiological guidelines put emphasis on conducting revascularization in the case of a 50% coronary stenosis, with confirmed myocardial ischaemia. It is known that severe myocardial ischaemia can occur in the absence of an obstructive coronary stenosis, depending on other mechanisms [31]. That is the principal disadvantage of morphological assessment, provided by coronarography. That is why, especially in patients with diabetes, the diagnostic methods should include assessment of myocardial perfusion (e.g. SPECT) and fractional flow reserve (FFR).

Although earlier surgical revascularization in diabetic patients could provide much benefit, the choice of appropriate candidates still remains problematic. As a consequence, different markers, allowing to identify high risk patients, are used in clinical practice.

However, new proposed markers may enlarge the diabetic group, in whom prompt revascularization could be more beneficial. Kleber et al. [32] suggested that BIO-VILCAD score including age, sex, left ventricular ejection fraction (EF), heart rate, N-terminal pro-brain natriuretic peptide, cystatin C, renin, 25OH-vitamin D3 and HbA1c, can be helpful in predicting the risk of long-term mortality in diabetics [32].

## Percutaneous intervention vs. surgical revascularization

Recently published guidelines, based on the results of widely available meta-analyses and large clinical trials, including BARI-2D, SYNTAX, CARDia, FREEDOM, allow us to compare both types of revascularization-percutaneous and surgical- in diabetics [30]. According to these recommendations, CABG remains the therapeutic strategy of choice for multivessel or complex (SYNTAX score >22) CAD in diabetic people. A considerable amount of evidence indicates that PCI is associated with a higher risk of repeat revascularization. On the other hand, CABG is connected with a higher risk of stroke. Nevertheless, FREEDOM trial [33] proved that all-cause mortality and myocardial infarction rate were significantly lower in the case of surgical revascularization, despite preserved current pharmacological regimens and drug eluting stents (DES) usage. The inferiority of PCI in multivessel or complex CAD was claimed to be dependent on the usage of a previous stent generation. In order to exclude the influence of a stent type, two large trials were conducted. According to the BEST trial, in patients with diabetes, the prevalence of a primary composite end point was significantly higher in the group treated with PCI, than in patients assigned to CABG [34]. The prevalence of both target-vessel and new lesion repeat revascularization, as well as the incidence of a major secondary composite end point, was higher in the PCI group, in a 2-year follow-up [34]. The significance maintained in the long-term follow-up [34]. In the second trial, Bangalore et al. [35] showed a better outcome for PCI in a 30-day observation, in respect of a lower mortality and stroke incidence. The difference between stroke incidence in the PCI and CABG group was no longer significant after 30-days from the index procedure. The mortality rate also became similar in both groups, in the long-term follow-up. However, the prevalence of myocardial infarction, especially spontaneous, and repeat revascularization was significantly higher for patients, treated with PCI. The difference was noted already in the short-term follow-up, and maintained. These post-procedural complications, particularly concerned patients with incomplete revascularization [35]. In an earlier study, comparing both strategies of revascularization in patients with resistant angina and high operative risk (EF < 35%, reoperation, MI < 7 days, IABP, age > 70 years), no difference was revealed [36].

Despite the choice of a therapeutic intervention, a prompt diagnosis of cardiac ischemia is the crucial factor, that could improve the clinical outcome in diabetics. An early CAD diagnosis would facilitate the complete, arterial revascularization. We suppose that accurate revascularization, in addition to a continuous, satisfying glycaemic control and pharmacotherapy, can improve the poor prognosis of a diabetic patient. Accurate revascularization, meaning by-passing right lesions (corresponding with an ischemic segment) and all lesions, using preferably arterial grafts.

## Improving the outcome of surgical revascularization

Data demonstrates that complete arterial revascularization improves clinical outcomes in diabetic subjects. Hoffman et al. [37] reported a lower 30-day mortality in a group of patients after arterial revascularization. This particularly beneficial effect was even higher after 10- and 12-years. These findings were supported by Agrifoglio et al. [38], who showed that revascularization with bilateral internal thoracic arteries (BITA) is beneficial in diabetics. In addition, BITA harvesting did not significantly increase the risk of mediastinitis [38]. Elsewhere, Hemo et al. [39] reported short- and long-term clinical outcome in people with diabetes, receiving BITA grafts. He noted that sternal infection rate was 3.7%, and this serious complication was strongly associated with reoperation, peripheral vascular disease, chronic lung disease, obesity and female sex. However, significantly improved survival, over 8.4 ± 4 years, outweighed the risk of wound infection. Additionally, it is suggested that skeletonization of the internal thoracic artery (ITA) can decrease the risk of wound infection and sternal dehiscence [40]. This fact is reflected in recent myocardial revascularization guidelines, in which ITA skeletonization is recommended in patients with diabetes [30].

It is postulated that surgical revascularization in diabetics should be performed without cardiopulmonary by-pass (CPB) support [39, 41, 42]. Results of an observational study demonstrate that less (both non- and cardiovascular) complications occur after a surgery, in the case of performing off-pump coronary artery bypass grafting (OPCAB) [30]. Emmert et al. [42] emphasised that the incidence of complete revascularization is not influenced by off-pump performance, but the choice of a final strategy depends mainly on the experience of a surgeon. During off-pump myocardial revascularization, techniques not involving aortic manipulations should be preferred, to reduce the incidence of neurological complications, and improve outcome.

## Glycaemic control in the perioperative period

A good glycaemic control is a particularly important factor, deciding the final effect of revascularization. A HbA1c level ≥9% should disqualify the patient from an elective surgical procedure and some authors recommend even HbA1c l ≥8% as a borderline value [43]. According to recent recommendations, the glycaemia should not exceed 180 mg/dL (10 mmol/L) during the perioperative period [44]. There is proof that a strict glycaemic control (90–120 mg/dL), in patients after CABG, does not improve the survival [45]. On the other hand, some investigators found the intensive insulinotherapy to improve cardiac output after CABG [46]. The postoperative, good metabolic control, significantly contributes to a longer graft patency in diabetics. It was shown that in people with diabetes and HbA1c >7.5%, the internal thoracic artery wall was oedemic and thicker [47].

## Influence of diabetes on antiplatelet drugs action

Despite surgical treatment and good management of hyperglycaemia, the risk of in-stent restenosis (ISR), graft occlusion or de novo stenosis remains higher in diabetics than in the general population. The higher risk of ISR in diabetics is associated with several well-recognized risk factors, including a higher prevalence of resistance to antiplatelet drugs, especially aspirin and clopidogrel [48]. The results of Angiolillo et al. [48] revealed that the altered metabolism of clopidogrel in diabetics, disturbs the drug activation. It is suggested that the administration of an activated metabolite or prasugrel/ticagrelor could resolve this problem.

## Future directions of treatment

Finally, it’s worth mentioning the concepts of decreasing the resistin levels, that would eliminate the principal cause of cardiovascular disorders in diabetics, aiming at inflammation, angiogenesis, endothelial dysfunction, altered lipids and glucose metabolism [16]. Thiazolidinediones are commonly available drugs, that reduce resistin levels. However, thiazolidinediones have their adverse effect on the cardiac function and should be avoided in HF [16]. Still the concept of reducing resistin levels is very encouraging, and should be studied.

## Conclusions

Diabetes and coronary artery disease are very often associated. Therefore, a good glycaemic control is a significant part at every stage of treatment in people with diabetes and cardiovascular diseases. It is suggested that chronic myocardial ischaemia underlies progressive diabetic cardiomyopathy, and untreated, inevitably leads to congestive heart failure. Concluding, at the moment the best way to prevent the progression of diabetic cardiomyopathy to heart failure, is to consider surgical revascularization in any diabetic patient diagnosed with CAD.

## Abbreviations

ACE-I: angiotensin-converting-enzyme inhibitor
ARBs: angiotensin receptor blockers
AF: atrial fibrillation
BITA: bilateral internal thoracic arteries
CABG: coronary artery by-pass grafting
CAD: coronary artery disease
CPB: cardio-pulmonary bypass
DC: diabetic cardiomyopathy
DES: drug eluting stents
EASD: European Association for the Study of Diabetes
EF: left ventricle ejection fraction
FFA: free fatty acids
FFR: fractional flow reserve
HA: arterial hypertension
HF: heart failure
IABP: intraaortic balloon pump
ISR: in-stent restenosis
ITA: internal thoracic artery
LAD: left anterior descending artery
LAVI: left atrium volume index
LMS: left main stem
MI: myocardial infarction
NSTEMI: non-ST segment elevation myocardial infarction
OPCAB: off-pump coronary artery by-pass grafting
PCI: percutaneous coronary interventions
SPECT: single-photon emission computed tomography
T2DM: type 2 diabetes mellitus
WHO: World Health Organization
